# torchtree: Flexible Phylogenetic Model Development and Inference Using PyTorch

**DOI:** 10.1093/sysbio/syaf047

**Published:** 2025-07-04

**Authors:** Mathieu Fourment, Matthew Macaulay, Christiaan J Swanepoel, Xiang Ji, Marc A Suchard, Frederick A Matsen IV

**Affiliations:** Australian Institute for Microbiology and Infection, University of Technology Sydney, 5 Broadway, Ultimo, NSW 2007, Australia; Australian Institute for Microbiology and Infection, University of Technology Sydney, 5 Broadway, Ultimo, NSW 2007, Australia; Centre for Computational Evolution, The University of Auckland, 303/38 Princes St, Auckland, 1010, New Zealand; School of Computer Science, The University of Auckland, 303/38 Princes St, Auckland, 1010, New Zealand; Department of Mathematics, Tulane University, 6823 St. Charles Avenue, New Orleans, LA 70118, USA; Department of Human Genetics, University of California, 885 Tiverton Drive, Los Angeles, CA 90095, USA; Department of Computational Medicine, University of California, 885 Tiverton Drive, Los Angeles, CA 90095, USA; Department of Biostatistics, University of California, 695 Charles E Young Dr. South, Los Angeles, CA 90095, USA; Public Health Sciences Division, Fred Hutchinson Cancer Research Center, 1100 Fairview Ave N, Seattle, WA 98109, USA; Department of Statistics, University of Washington, Padelford Hall, Northeast Stevens Way, Seattle, WA 98195, USA; Department of Genome Sciences, University of Washington, 3720 15th Ave NE, Seattle, WA 98195, USA; Howard Hughes Medical Institute, Fred Hutchinson Cancer Research Center, 1100 Fairview Ave N, Seattle, WA 98109, USA

**Keywords:** Bayesian inference, phylogenetics, PyTorch, variational Bayes

## Abstract

Bayesian inference has predominantly relied on the Markov chain Monte Carlo (MCMC) algorithm for many years. However, MCMC is computationally laborious, especially for complex phylogenetic models of time trees. This bottleneck has led to the search for alternatives, such as variational Bayes, which can scale better to large data sets. In this paper, we introduce torchtree, a framework written in Python that allows developers to easily implement rich phylogenetic models and algorithms using a fixed tree topology. One can either use automatic differentiation or leverage torchtree’s plug-in system to compute gradients analytically for model components for which automatic differentiation is slow. We demonstrate that the torchtree variational inference framework performs similarly to BEAST in terms of speed, and delivers promising approximation results, though accuracy varies across scenarios. Furthermore, we explore the use of the forward Kullback–Leibler (KL) divergence as an optimizing criterion for variational inference, which can handle discontinuous and nondifferentiable models. Our experiments show that inference using the forward KL divergence is frequently faster per iteration compared with the evidence lower bound (ELBO) criterion, although the ELBO-based inference may converge faster in some cases. Overall, torchtree provides a flexible and efficient framework for phylogenetic model development and inference using PyTorch.

Markov chain Monte Carlo (MCMC) has been the engine of Bayesian inference in phylogenetics over the last 20 years. It is widely used (Ronquist et al. 2012; Höhna et al. 2016; Suchard et al. 2018) and is considered the gold standard because once converged, it samples the posterior distribution exactly (although with autocorrelation). However, MCMC is best suited to smaller data sets because it is computationally laborious, especially for complex models centered around time trees. Due to this computational barrier, researchers with large data sets often opt for non-Bayesian methods based on maximum likelihood (Sagulenko et al. 2018), parsimony (Turakhia et al. 2021), and other heuristics (Tamura et al. 2012; To et al. 2016), although these methods are not amenable to complex model inference and, unlike Bayesian inference, deriving and interpreting confidence intervals for estimates can be difficult.

Variational Bayes (VB), also called variational inference, is an alternative approach for Bayesian inference that can scale better to large data sets (Jordan et al. 1999). VB uses optimization to find the closest approximation, the *variational distribution*, to the posterior from a family of densities. Consequently, it can be faster than MCMC, although it is not guaranteed to provide an exact representation of the posterior. One can choose from several criteria to define closeness, but the Kullback–Leibler (KL) divergence is the most common.

The optimization process in VB algorithms, such as the automatic differentiation variational inference algorithm (Kucukelbir et al. 2017), requires the gradient of the posterior with respect to the model parameters. If the model is complex, computing this gradient efficiently is especially important, either analytically or with automatic differentiation. Libraries that implement automatic differentiation algorithms such as PyTorch (Paszke et al. 2019), TensorFlow (Abadi et al. 2016), Stan Math Library (Carpenter et al. 2015), and JAX (Bradbury et al. 2018) calculate these gradients for the optimizer. PyTorch, TensorFlow, and JAX are Python-based libraries that offer user-friendly interfaces for high-level programming backed by efficient compiled code under the hood to accelerate computations.

Fourment and Darling (2019) introduced phylostan, which was the first package that used automatic differentiation to approximate phylogenetic models using variational inference and hamiltonian Monte Carlo (HMC) (Neal 2011). This Python program generates phylogenetic models in the Stan language, which are fed to the Stan program (Carpenter et al. 2017). The Stan team developed their own language, which makes use of the Stan Math Library (Carpenter et al. 2015), a C++ library, for automatic differentiation. It works with a single fixed topology with a wide range of phylogenetic models. Although creating new models is straightforward thanks to the ease of learning the Stan language, our benchmark on phylogenetic models indicated that Stan programs were slower than PyTorch (Fourment et al. 2023), likely due to differences in the implementation of their automatic differentiation frameworks.

In the context of variational inference, optimization in Stan is limited to a single objective—the evidence lower bound (ELBO; described later)—using either fully factorized univariate Gaussian distributions or a single multivariate Gaussian distribution as variational distributions, leveraging the automatic differentiation variational inference framework (Kucukelbir et al. 2017). Adding additional objectives or variational distributions is difficult because extending the code base is tedious as it requires a deep understanding of the C++ code base. Initializing starting values for the variational distribution in a Stan program is challenging, and using random values often results to convergence issues (Fourment and Darling 2019).

VB has garnered increasing attention lately, as evidenced by a surge in related research papers (Dang and Kishino 2019; Liu et al. 2021; Moretti et al. 2021; Ki and Terhorst 2022; Koptagel et al. 2022; Swanepoel et al. 2022; Zhang and Matsen 2024). A major difficulty in applying VB to phylogenetic modeling is dealing with the topology, the discrete component of the model.

It is tempting to implement a phylogenetic package for VB that relies exclusively on efficient analytical gradient calculations, either by starting from scratch or by building upon established software such as BEAST (Suchard et al. 2018). Early studies (Fourment and Darling 2019; Fourment et al. 2020, 2023) showed significant improvements in terms of computational efficiency with such software at the cost of implementing the gradient of every model component. Specifically, our previous benchmarking results (Fourment et al. 2023) suggested a strategy in which the computationally expensive parts of gradient computation (such as the phylogenetic likelihood) are computed with specialized algorithms (Ji et al. 2020), whereas less expensive model (e.g., parametric prior distributions) gradient calculations are performed using automatic differentiation. Although hand-crafted optimizations for specific models provide computational efficiency, they can limit extensibility and increase maintenance costs, making it harder to adapt models to new inference techniques.

Thus, overall, we feel that it is time for the community to have a package that gets the best of both worlds: fast hand-crafted gradients for the tree likelihood function and automatic differentiation for the prior distributions—as well as for variational distributions in the context of variational inference. By leveraging automatic differentiation, we facilitate model extensions and reduce the overhead associated with manually deriving and maintaining gradients, thereby enhancing long-term scalability and reproducibility. We are inspired by progress in deep learning, where the development of easy-to-use packages such as PyTorch has led to an explosion of diverse model architectures.

We propose torchtree, a framework written in Python, a language widely used by researchers, that allows developers to easily implement phylogenetic models and algorithms. Inference is carried out under the assumption of a fixed tree topology, a common consideration in fast programs designed for large-scale phylodynamic analysis (To et al. 2016; Hadfield et al. 2018; Sagulenko et al. 2018). torchtree is primarily focused on variational inference; other algorithms such as MCMC, HMC, and maximum a posteriori inference are implemented. It is already significantly faster than phylostan when using automatic differentiation, and we provide several plug-ins supplying analytic gradients that significantly improve its speed as shown in other studies (Fourment and Darling 2019; Fourment et al. 2023).

Some adjustments need to be made in transitioning from an MCMC-based approach to a VB approach, leveraging automatic differentiation. For example, discontinuous models such as the skygrid (Gill et al. 2013) may cause problems for gradient-based approaches. To explore this in a case study, we propose a piecewise-linear adaptation of the skygrid which we call the *skyglide*. Although this model is not differentiable, it is continuous and thus more regular than the skygrid.

In contrast to MCMC where the Metropolis–Hastings algorithm is universal, VB objectives are more diverse and require consideration. We explore those here, including the ELBO (which requires the gradient of the posterior with respect to the variational parameters) and the forward KL divergence (which does not require the gradient). We find that ELBO-based variational inference performs poorly on skygrid coalescent models, presumably due to the discontinuities present in the piecewise-constant population size function. By using the forward KL divergence as the optimizing criterion, we can circumvent this continuity issue, as it does not require the model to be continuous or differentiable. Our results also indicate that inference using the forward KL divergence is frequently faster per iteration compared with those using the ELBO; however, in some cases the ELBO-based inference converges faster than the forward KL-based method. In some cases, the approximation with the forward KL was just as accurate as the ELBO-based approximation. However, we were surprised to find that the forward KL did not better estimate uncertainty relative to the ELBO. We also show that even though the skyglide model is not differentiable, it performs well under ELBO-based inference.

## Materials and Methods

### Variational Inference

Variational inference seeks to minimize a measure $\mu$ between the posterior and a simpler variational distribution chosen from a family of distributions. For phylogenetic inference, the posterior probability of a tree’s continuous parameters $\boldsymbol {z} \in Z$ conditioned on a sequence alignment *D* with a fixed topology $\tau$ is $p(\boldsymbol {z}|D, \tau )$. The variational distribution $q(\boldsymbol {z}; \boldsymbol {\phi })$ is defined over the tree’s continuous variables and is parameterized by $\boldsymbol {\phi }$. The objective is to find the optimal variational approximation from a family of functions $q(\boldsymbol {z}) \in \mathcal {Q}$:


\begin{eqnarray*}
q^{*}(\boldsymbol {z}) = {\rm argmin}_{q(\boldsymbol {z}; \boldsymbol {\phi }) \in \mathcal {Q}} \mu (q(\boldsymbol {z}; \boldsymbol {\phi }) || p(\boldsymbol {z}|D, \tau )).
\end{eqnarray*}

The solution found through optimization $q^{*}$ then serves as an approximation for the posterior distribution. Typically, practitioners use the backward KL divergence for the measure between two distributions $\mu (q, p) = \operatorname{KL}(q||p)$ (Blei et al. 2017). It measures the amount of information lost by using the approximation $\operatorname{KL}(q||p) = \mathbb {E}_{q(\boldsymbol {z}; \boldsymbol {\phi })}[\log q(\boldsymbol {z}; \boldsymbol {\phi })] - \mathbb {E}_{q(\boldsymbol {z}; \boldsymbol {\phi })} [\log p(\boldsymbol {z}| D, \tau )]$. Minimizing KL divergence is equivalent to maximizing a lower bound of the evidence called the ELBO:


\begin{eqnarray*}
\mathcal {L}(q) = \mathbb {E}[\log (p(\boldsymbol {z}, D | \tau ))] - \mathbb {E}[\log (q(\boldsymbol {z}; \boldsymbol {\phi }))],
\end{eqnarray*}

where the expectations are taken with respect to the variational distribution *q*. Informally, we choose the parameters $\boldsymbol {\phi }$ of the variational distribution *q* via numerical optimization such that the variational distribution is as close as possible to the posterior distribution. This comes by expanding the second term of the KL divergence using Bayes’ rule and dropping the intractable quantity $p(D, \tau )$, which is a constant, making the computation of the ELBO relatively inexpensive.

Another choice of measure is the forward KL divergence


\begin{eqnarray*}
\operatorname{KL}(p||q) = \mathbb {E}_{p(\boldsymbol {z}| D, \tau )}[\log p(\boldsymbol {z}| D, \tau ) - \log q(\boldsymbol {z}; \boldsymbol {\phi })].
\end{eqnarray*}

This version reflects the “intent” of the KL divergence, with the “ground truth” being the first term. In theory, its mass-covering behavior should not result in underdispersed approximations of the distribution of interest (Naesseth et al. 2020; Jerfel et al. 2021), although it can be computationally intractable. This is in contrast to the mode-seeking behavior observed when optimizing $\operatorname{KL}(q||p)$, which frequently underestimates the variance of the posterior (see the “Mass-Covering vs. Mode-Seeking: Forward vs. Reverse KL” section in Supplementary Material for a visual explanation).

However, directly minimizing this measure poses challenges as it necessitates sampling from the posterior distribution. We can use a self-normalized importance sampling (SNIS) gradient estimator (Bornschein and Bengio 2015; Jerfel et al. 2021) to estimate this measure and its gradient. The importance sampling estimate of $\operatorname{KL}(p||q)$ using the instrument distribution *q* is


\begin{eqnarray*}
\operatorname{KL}(p||q) & = \mathbb {E}_{p(\boldsymbol {z}| D, \tau )}\log \left[\frac{p(\boldsymbol {z}| D, \tau )}{q(\boldsymbol {z}; \boldsymbol {\phi })}\right]\\
& = \mathbb {E}_{q(\boldsymbol {z}; \boldsymbol {\phi })} \left[\frac{p(\boldsymbol {z}| D, \tau )}{q(\boldsymbol {z}; \boldsymbol {\phi })} \log \frac{p(\boldsymbol {z}| D, \tau )}{q(\boldsymbol {z}; \boldsymbol {\phi })} \right] \\
& = \frac{\mathbb {E}_{q(\boldsymbol {z}; \boldsymbol {\phi })} \left[\frac{p(\boldsymbol {z}, D, \tau )}{q(\boldsymbol {z}; \boldsymbol {\phi })} \log \frac{p(\boldsymbol {z}| D, \tau )}{q(\boldsymbol {z}; \boldsymbol {\phi })} \right]}{\mathbb {E}_{q(\boldsymbol {z}; \boldsymbol {\phi })} \left[\frac{p(\boldsymbol {z}, D, \tau )}{q(\boldsymbol {z}; \boldsymbol {\phi })} \right]} \\
& \approx \sum _{s=1}^S \log \left(\frac{p(\tilde{\boldsymbol {z}}_s| D, \tau )}{q(\tilde{\boldsymbol {z}}_s ; \boldsymbol {\phi })}\right) w_s ,
\end{eqnarray*}

where $\tilde{\boldsymbol {z}}_s \sim q(\boldsymbol {z}; \boldsymbol {\phi })$ and


\begin{eqnarray*}
w_s = \frac{p(\tilde{\boldsymbol {z}}_s, D, \tau )}{ q(\tilde{\boldsymbol {z}}_s; \boldsymbol {\phi })} \Big / \sum _{i=1}^N \frac{p(\tilde{\boldsymbol {z}}_i, D, \tau )}{q(\tilde{\boldsymbol {z}}_i; \boldsymbol {\phi })}.
\end{eqnarray*}

An importance sampling estimate of the gradient of the $\operatorname{KL}(p||q)$ divergence can be computed similarly as follows. Notice that $\mathbb {E}_{p(\boldsymbol {z}| D, \tau )}[\log p(\boldsymbol {z}| D, \tau )]$ does not depend on $\phi$; therefore, minimizing $\operatorname{KL}(p||q)$ is equivalent to minimizing an objective $L_{\operatorname{KL}}(\phi )$ based on cross-entropy with respect to variational parameters,


\begin{eqnarray*}
L_{\operatorname{KL}}(\phi ) = - \mathbb {E}_{p(\boldsymbol {z}| D, \tau )} [\log q(\boldsymbol {z}; \boldsymbol {\phi })].
\end{eqnarray*}

Thus, we can first take the gradient and then use the SNIS estimator, obtaining


\begin{eqnarray*}
\nabla L_{\operatorname{KL}}(\phi ) = -\sum _{s=1}^S w_s \nabla \log q(\tilde{\boldsymbol {z}}_s ; \boldsymbol {\phi }) , \quad \tilde{z}_s \sim q(\boldsymbol {z}; \boldsymbol {\phi }).
\end{eqnarray*}

Estimation of the gradient with respect to the parameters of *q* can have high variance because we optimize over the same distribution from which samples are drawn. Another source of error is that the number of samples *S* required for SNIS to provide accurate mean estimates scales exponentially with $\operatorname{KL}(p||q)$ (Chatterjee and Diaconis 2018).

The reverse KL divergence $\mathrm{KL}(q || p)$ minimizes the discrepancy from the approximate distribution $q(\boldsymbol {z})$ to the true posterior $p(\boldsymbol {z} \mid D)$. Because it heavily penalizes assigning probability to regions where *q* has mass but *p* does not, it tends to be *mode-seeking*, favoring a sharper approximation around the dominant mode. In contrast, the forward KL divergence $\mathrm{KL}(p || q)$ minimizes the divergence in the opposite direction and should strongly penalize regions where *p* has mass but *q* does not, leading to a *support-covering* behavior, where *q* spreads to capture more of *p*, even if it means overestimating uncertainty.

A key advantage of using $\operatorname{KL}(p||q)$ is that the posterior does not have to be differentiable. Opting out of gradient calculation can enhance computational efficiency, as the computational expense of gradient calculations is typically high. The gradient calculation using reverse-mode automatic differentiation typically incurs a small constant factor overhead over the function evaluation (Margossian 2019). However, we have shown that for the branch length and substitution model parameters, the gradient calculation can be up to 8× slower than tree likelihood evaluation (Fourment et al. 2023). In practice, employing the variational distribution as the importance distribution in the SNIS algorithm typically reduces the approximation variance.

Choosing an appropriate family of approximating distributions is crucial for balancing computational efficiency and accuracy. Two common choices are the mean-field approximation and the full-rank approximation.

The mean-field approximation assumes that the variational distribution factorizes completely across all latent variables such as


\begin{eqnarray*}
q(\boldsymbol {z}; \boldsymbol {\phi }) = \prod _i q(z_i; \phi _i).
\end{eqnarray*}

Mean-field approximations tend to be computationally efficient but may provide overconfident estimates due to their inability to capture dependencies in the true posterior. In contrast, the full-rank approximation allows for dependencies between latent variables by using a Gaussian distribution with a full covariance matrix:


\begin{eqnarray*}
q(\boldsymbol {z}; \boldsymbol {\phi }) = \mathcal {N}(\boldsymbol {\mu }, \boldsymbol {\Sigma }).
\end{eqnarray*}

It is common practice to reparameterize the covariance matrix using a Cholesky factorization, $\boldsymbol {\Sigma } = \boldsymbol {L} \boldsymbol {L}^\intercal$, and the variational distribution becomes $q(\boldsymbol {z}; \boldsymbol {\phi }) = \mathcal {N}(\boldsymbol {\mu }, \boldsymbol {L} \boldsymbol {L}^\intercal )$ (Kucukelbir et al. 2017).

The full-rank approach can better approximate complex posterior shapes, as it captures correlations between parameters. However, it is significantly more computationally expensive, particularly when the number of latent variables is large.

### Design and Implementation of torchtree

torchtree implements a variety of Bayesian inference techniques, including maximum a posteriori optimization, variational inference, and HMC. It also provides out-of-the-box extensions to variational inference including multisampling, normalizing flows, and various divergence measures. It implements a wide range of phylogenetic models (Table 1), allowing investigating complex phylodynamic questions. To demonstrate this functionality, we introduce some implementation details.

**Table 1.** Base models implemented in torchtree

Type	Model
Nucleotide substitution model	Standard reversible models and SRD06
Amino acid substitution model	WAG and LG
Codon model	MG94
Rate heterogeneity across site	Proportion of invariant sites, Weibull
Phylogeography	Discrete
Birth death	Constant, BDSKY
Coalescent	Constant, exponential, skyride, skygrid, skyglide
Clock prior	Clock-free, strict, autocorrelated, uncorrelated

torchtree is implemented in Python and uses PyTorch to leverage automatic differentiation. Its design is inspired by the BEAST packages (Suchard et al. 2018; Bouckaert et al. 2019), specifically the object structure, the plug-in architecture, and the file formats. In torchtree, the specification of model and algorithm parameters are specified through JSON files, whereas BEAST uses the XML format. Much like BEAST2, torchtree provides a simple framework for creating plug-ins that can extend without modifying the existing code base.

torchtree features a plug-in system that allows users to add functionality. For example, in some cases some functions can be implemented more efficiently than with automatic differentiation. Indeed, Fourment and Darling (2019) and Fourment et al. (2023) showed that analytical derivatives in a compiled language generally outperform those derived through automatic differentiation. Mixing automatic differentiation and compiled analytic derivative code is possible with custom C++ extensions provided by PyTorch. We describe later in this section torchtree plug-ins that make use of these custom extensions.

A simple command-line interface is provided in order to build configuration files. However, the user will need to edit it manually in order to adjust parameters such as hyperprior parameters. Documentation is provided using Python docstrings in order to understand the building blocks. torchtree is open source software, available on GitHub at https://github.com/4ment/torchtree, along with compiled API documentation. Examples of usage can be seen at https://github.com/4ment/torchtree-experiments.

#### Plug-ins

torchtree provides a plug-in system that allows users and developers to implement new models and algorithms. In its current form, torchtree is implemented entirely in Python, with no need for the user to install any plug-ins.

#### torchtree-physher

The torchtree-physher plug-in uses physher (Fourment and Holmes 2014), a C-based program, to efficiently evaluate several likelihood functions and their gradients (Table 2). Every model implemented in torchtree-physher is analytically differentiable, except the gamma site model for which the gradient is approximated numerically. The Weibull site model, suggested by Fourment and Darling (2019), serves as an alternative to the widely used gamma site model (Yang 1994). It is favored for its closed-form inverse cumulative distribution function (CDF), allowing straightforward analytical gradient calculation. The derivatives with respect to the branch lengths are efficiently calculated using a linear-time algorithm (Fourment et al. 2020), resulting in a substantial speed boost in a benchmark between physher and phylostan (Fourment and Darling 2019). The gradient of the Jacobian transform of the node height reparameterization (Fourment and Darling 2019) is efficiently calculated using the method proposed by Ji et al. (2021).

**Table 2.** Models implemented in torchtree-physher

Type	Model
Nucleotide model	JC69, HKY, GTR and SRD06
Amino acid model	WAG and LG
Rate heterogeneity across site	Proportion of invariant sites, Weibull, gamma
Phylogeography	Discrete
Coalescent	Constant, skyride, skygrid, skyglide
Clock rate	Clock-free, strict, one rate per branch

#### torchtree-bito

torchtree-bito is a torchtree plug-in that offers an interface to the bito library (https://github.com/phylovi/bito). Within bito, analytical derivatives with respect to the branch lengths and the parameter of the Weibull site model are calculated through the BEAGLE library (Ayres et al. 2019; Ji et al. 2020). The gradient with respect to the GTR substitution model parameters is calculated numerically using finite differences. BEAGLE also provides efficient calculation of the tree likelihood and its gradient on graphics processing units (GPUs) (Gangavarapu et al. 2024).

#### torchtree-scipy and torchtree-tensorflow

The discretized gamma distribution is widely used to model rate heterogeneity across sites. The discretization method requires the inverse CDF of the gamma distribution, which, at the time of writing (version 2.2.1), is not available in PyTorch. torchtree-scipy and torchtree-tensorflow are simple plug-ins that implement the gamma-distributed site model using the SciPy and TensorFlow libraries, respectively. Although torchtree-tensorflow calculates gradients using automatic differentiation, torchtree-scipy approximates them using central finite differences.

### A Continuous Piecewise-Linear Coalescent Model

We have introduced a coalescent model analogous to the piecewise-constant model on a fixed grid (also known as skygrid) (Gill et al. 2013) but instead with a piecewise-linear ancestral population size. The model, which we call “skyglide,” is parameterized in terms of ancestral population sizes at times on a fixed grid, and interpolates between them using linear functions. Specifically, we take


\begin{eqnarray*}
p(t_2, \dots , t_{n+1} \mid N(t)) &=& \prod _{k=2}^{n} p(t_{k} \mid t_{k+1}, N(t))\\
&=& \prod _{k=2}^n {k \atopwithdelims ()2} \frac{1}{N(t_{k})}\\
&&\times \,\exp \left[ -\int _{t_{k+1}}^{t_k} {k \atopwithdelims ()2} \frac{1}{N(t)} dt \right].
\end{eqnarray*}

Let $\boldsymbol \theta = (\theta _0, \dots , \theta _M)$ be the vector of effective population sizes at fixed and equidistant time points $0=x_0, \dots , x_M=C$, where *C* is the user-defined cutoff value of the grid. Then, we define the piecewise-linear demographic function,


\begin{eqnarray*}
\widehat{N}(t) = \left\lbrace \begin{array}{@{}l@{\quad }l@{}}\theta _i + (\theta _{i+1} - \theta _i) \frac{t - x_i}{x_{i+1} - x_i} & \text{if } x_i \le t \le x_{i+1} \\
\theta _M & \text{if } t > x_M \end{array}\right..
\end{eqnarray*}

This is in contrast to the piecewise-constant function, which assumes $\widehat{N}_c(t) = \theta _i$ for $x_i \le t < x_{i+1}$ and $\widehat{N}_c(t) = \theta _M$ for $t > x_M$.

Each subfunction of $\widehat{N}(t)$ and $\widehat{N}_c(t)$ is continuously differentiable because it is either a linear or constant function. The key difference between the two piecewise functions is that $\widehat{N}_c(t)$ contains jump discontinuities, whereas $\widehat{N}(t)$ is continuous across its domain (see the “Continuity and differentiability of piecewise-linear coalescent model” section in Supplementary Material). Although neither of the piecewise functions is differentiable, our analyses indicate that, unlike the skygrid model, the skyglide model can be effectively utilized with gradient-based algorithms.

We show that, much like the piecewise-constant model, the piecewise-linear model can successfully reconstruct effective population size trajectories under three simulated demographic scenarios (see the “Validation of piecewise-linear coalescent model” section in Supplementary Material and Figs. S11–S13). These findings are consistent with those of Billenstein and Höhna (2024), who also observed virtually no difference between piecewise-constant and piecewise-linear skyline models when using a change-point method estimated via MCMC.

### Data Sets and Validation

We analyzed two data sets to exemplify common and new features implemented in torchtree, as well as to illustrate the behavior of various objectives for different types of models.

The first data set comprises 63 RNA sequences of type 4 from the E1 region of the hepatitis C virus (HCV) genome that were isolated in 1993. As in previous studies (Pybus et al. 2001), the substitution rate was fixed to $7.9 \times 10^{-4}$ substitutions per site per year. As torchtree can only accommodate a single topology, we also enforce the constraint of a fixed tree topology in all BEAST analyses. The topology used in the torchtree and BEAST analyses was drawn randomly from a sample of trees generated by a preliminary analysis with BEAST without topological constraints. We used the GTR substitution model and gamma distributed rate heterogeneity with four categories. For this analysis, torchtree-physher plug-in was used to compute gradients for the tree likelihood. We used either a piecewise-constant (also known as skygrid) (Gill et al. 2013) or piecewise-linear (skyglide) population size coalescent prior with a cutoff of 400 years and 75 time segments. As is customary for piecewise-constant coalescent models (Minin et al. 2008; Gill et al. 2013), we place a Gaussian Markov random field (GMRF) prior on the vector of log effective population sizes and a gamma prior with rate and scale equal to 0.005 on the precision parameter. For unconstrained optimization, node heights are reparameterized using the approach outlined by Fourment and Darling (2019). We evaluated both the mean-field and full-rank approximations using two criteria: ELBO and $\operatorname{KL}(p||q)$ and optimized them for 10 million iterations. As reported by Fourment and Darling (2019), the full-rank approximation is highly sensitive to initialization, requiring well-chosen starting values to avoid stability issues with the covariance matrix. One way to mitigate this issue is by using a much smaller learning rate ($10^{-5}$) compared with the mean-field analysis (0.1), but this can slow convergence or even prevent the algorithm from fully optimizing. To improve stability, we initialized the full-rank approximation using the optimized parameter estimates from the mean-field distribution. The off-diagonal covariances were initialized to 0, making the full-rank distribution identical to the optimized mean-field distribution. The gradient of the ELBO is calculated using a single sample (Kucukelbir et al. 2017), whereas the gradient of the forward KL divergence is calculated using the SNIS estimator with 10 samples. We also performed HMC inference with torchtree to approximate the model with the skyglide model for 50 million iterations. The step size of the leap frog integrator and the diagonal mass matrix were tuned automatically. Every model was approximated using 50 million MCMC iterations in BEAST.

The second data set is made of 583 SARS-CoV-2 RNA sequences from Pekar et al. (2021). Using a fixed topology, we replicated the analyses of Magee et al. (2024) to assess whether there is a rate increase of C$\rightarrow$T substitutions over the reverse T$\rightarrow$C substitutions. The rooted topology is obtained through a two-step process: first, by estimating the maximum likelihood (unrooted) tree using iqtree (Minh et al. 2020), and then by determining the root location using lsd (To et al. 2016). We implemented the HKY substitution model with random effects to allow for nonreversibility (see Magee et al. 2024 for more information on the model). Due to discontinuities in the skygrid model, we used a skyglide model with five parameters and a cutoff of 0.3 years. Although Magee et al. (2024) used a regularized Bayesian bridge prior (Nishimura and Suchard 2023), we opted to use the original Bayesian bridge formulation (Polson et al. 2013). The Bayesian bridge prior on random effect $\epsilon$ has density


\begin{eqnarray*}
p(\epsilon | \tau , \alpha ) \propto \exp \left(-\left|\frac{\epsilon }{\tau }\right|^\alpha \right).
\end{eqnarray*}

In this study, the exponent is fixed to $\alpha =0.25$ and we place a gamma prior on $\tau ^{-\alpha }$ with shape $\delta =1$ and scale $\theta =2$.

We can test the support for nonreversibilities, for example, the difference between the C$\rightarrow$T and T$\rightarrow$C rates, with Bayes factors. The fact that a model with the C$\rightarrow$T and T$\rightarrow$C rates equal (reversible with respect to C$\leftrightarrow$T) is nested within the random-effects model allows us to use the Savage–Dickey ratio (see Wagenmakers et al. 2010 for an example) to compute the Bayes factor from the posterior distribution of the random-effects model (detailed calculations are provided in the “Bayes factor calculation for mean-field variational inference” section of Supplementary Material).

As in the original study, we used the GTR substitution model to visually inspect the effect of using random effects in the substitution rate matrix. The GTR and HKY-RE models were approximated using 50 million MCMC iterations in BEAST. For the variational inference analyses, we optimized the ELBO for 1 million iterations.

In our analyses, we employed the mean-field approximation, modeling each factor as a univariate Gaussian distribution. To ensure that the phylogenetic parameters adhered to their respective constraints, we applied differentiable invertible transformations, aligning them with the support of the Gaussian distributions. For example, we used the exponential function to transform positive parameters, and the logistic function to map parameters in the interval [0,1] onto the real line. These transformations, along with the mean-field approximation, are the foundations of the automatic differentiation variational inference framework (Carpenter et al. 2017; Kucukelbir et al. 2017).

We investigated the speed of convergence of MCMC and variational inference using the coefficient of variation (CV) of the approximated distributions. We calculated the CV of each distribution at multiple time points and compared them with the CV of the final approximations. For an analysis of *M* iterations taking $t_M$ units of time, a time point indexed by its iteration number *i* is defined as $t_i=i \times t_M / M$. For MCMC, the coefficient of variation $CV(t_i)$ is calculated using samples from iteration 1 up to iteration *i*. With variational inference, we calculate the smoothed mean $\bar{\mu }(t_i)$ and variance $\bar{\sigma }^2(t_i)$ using the variational distributions from time 1 up to time $t_i$. We then compute the coefficient of variation $CV(t_i) = \sqrt{\bar{\sigma }^2(t_i)} / \bar{\mu }(t_i)$, where $\bar{\mu }(t_i) = \frac{\sum _{j=1}^i \mu (t_j)}{i}$ and $\bar{\sigma }^2(t_i) = \frac{\sum _{j=1}^i \sigma ^2(t_j)}{i}$. In the corresponding plots (Figs. S9 and S10), we show the relationship between $t_i$ for $\lbrace i\times 1000 | i=1,2,3,\dots ,M/1000\rbrace$ and the squared difference $(CV(t_i) - CV(t_M))^2$, showing how quickly the algorithms converge over time.

Given that the a priori choice of the number of iterations resulted in different runtimes for BEAST and torchtree, we use the same total runtime (i.e. $t_M$) for both. This approach is equivalent to having a consistent time budget constraint across the two methods.

Nextflow pipeline running every analysis is available from https://github.com/4ment/torchtree-experiments.

## Results

### Effective Population Size Estimation of HCV Data Set

**Figure 1.**
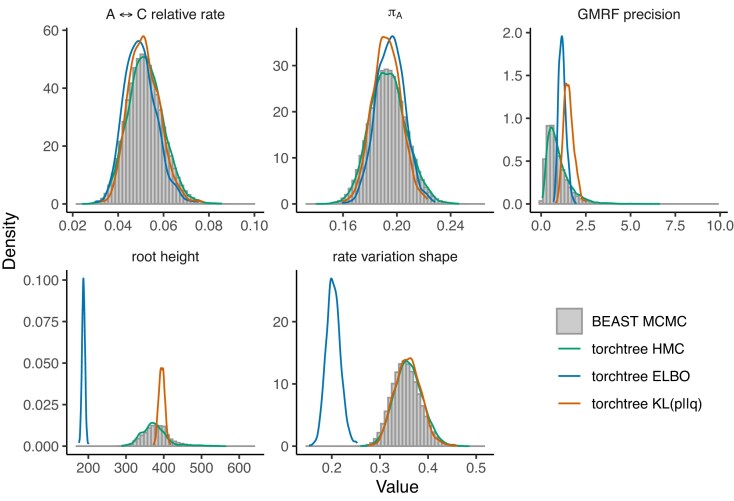
Posterior approximation of phylogenetic model parameters using torchtree and BEAST on the HCV data set with the skygrid (piecewise-constant) model. torchtree approximates the distributions using either mean-field variational inference [ELBO and $\operatorname{KL}(p||q)$] or HMC. BEAST uses MCMC. The plot displays density distributions for several parameters: the substitution rate bias between nucleotide A and C ($\mathrm{A} \leftrightarrow \mathrm{C}$), the frequency of nucleotide A ($\pi _{\mathrm{A}}$), the GMRF precision parameter, the age of the root node (root height), and the shape parameter of the discrete gamma site model. The gradient-based ELBO inference clearly struggles in this case of a discontinuous model.

**Figure 2.**
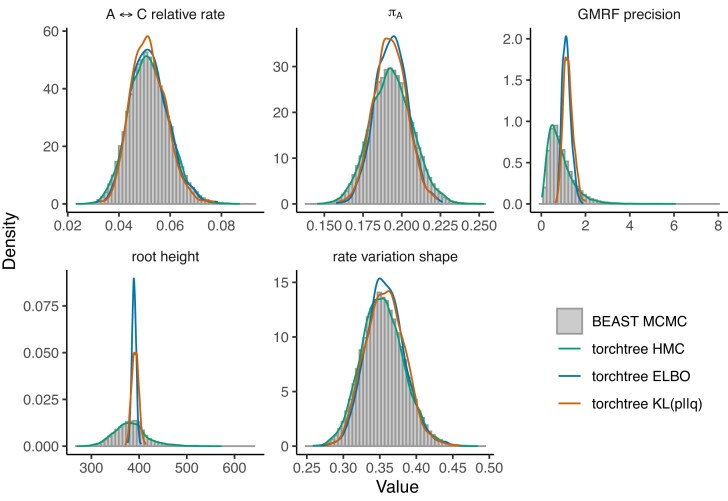
Posterior approximation of phylogenetic model parameters using torchtree and BEAST on the HCV data set with the skyglide (piecewise-linear) model. torchtree approximates the distributions using either mean-field variational inference [ELBO and $\operatorname{KL}(p||q)$] or HMC. BEAST uses MCMC. The plot displays density distributions for several parameters: the substitution rate bias between nucleotides A and C ($\mathrm{A} \leftrightarrow \mathrm{C}$), the frequency of nucleotide A ($\pi _{\mathrm{A}}$), the GMRF precision parameter, the age of the root node (root height), and the shape parameter of the discrete gamma site model.

First, we analyzed a data set consisting of HCV RNA sequences under two piecewise coalescent priors: piecewise-constant (skygrid) and piecewise-linear (skyglide) models. When utilizing the ELBO criterion and gradient-based optimization, it becomes evident that the discontinuous piecewise-constant model struggles to retrieve the root height and shape parameter of the gamma site model (Fig. 1), unlike the piecewise-linear model, which offers more accurate approximations (Fig. 2). In contrast, optimizing the $\operatorname{KL}(p||q)$ objective yields more accurate approximations of these parameters under both piecewise models.

We interpret these results as a consequence of the discontinuities present in the piecewise-constant model. Indeed, because the $\operatorname{KL}(p||q)$ objective does not require the calculation of the gradient of the coalescent, this suggests that gradient computations may be at fault. The existence of jump discontinuities in the piecewise-constant model may prevent gradient-based optimization methods from converging to the correct solution. In contrast, the piecewise-linear model used in this study is continuous because the end point of one segment is the initial point of the next segment.

**Figure 3.**
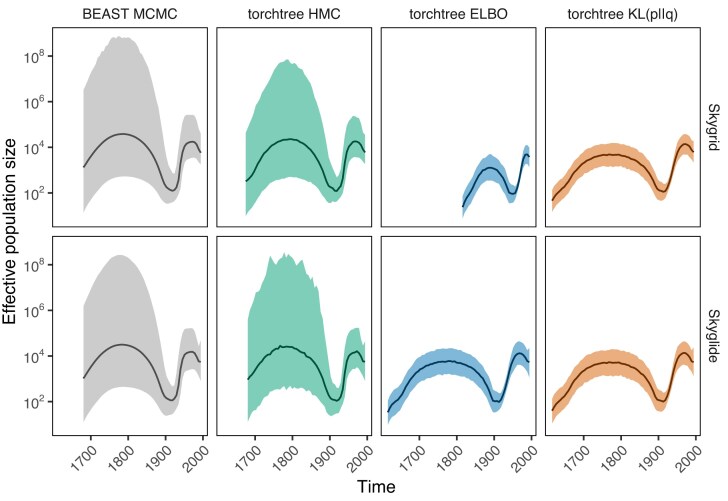
Posterior approximation of skyglide (piecewise-linear) and skygrid (piecewise-constant) population size distributions using torchtree and BEAST on the HCV data set.

These results are mirrored in the inference of ancestral population sizes (Fig. 3). All inference methods for the continuous skyglide model give compatible results, modulo smaller confidence intervals for the variational analysis as is common for this method. For the discontinuous skygrid model, the ELBO-based inference leads to a misestimation of the timescale of the demographic events.

The $\operatorname{KL}(p||q)$-based analysis was significantly faster than its ELBO counterpart, as the former method required 300 min, whereas the latter took 930 min. This was due to the fact that the ELBO-based analysis required the calculation of joint distribution gradients, which is computationally expensive.

The estimates obtained using the full-rank variational distribution are very similar to those from the mean-field approximation (Figs. S2–S4), exhibiting the same shortcomings with the discontinuous piecewise-constant model. Although the per-iteration runtime of the full-rank inference is slightly higher than that of the mean-field approach, as discussed in the “Materials and Methods” section, we had to initialize the full-rank analysis using the mean-field estimates, which made the use of the full-rank distribution more cumbersome.

### C to T Bias in SARS-CoV-2 Evolution

**Figure 4.**
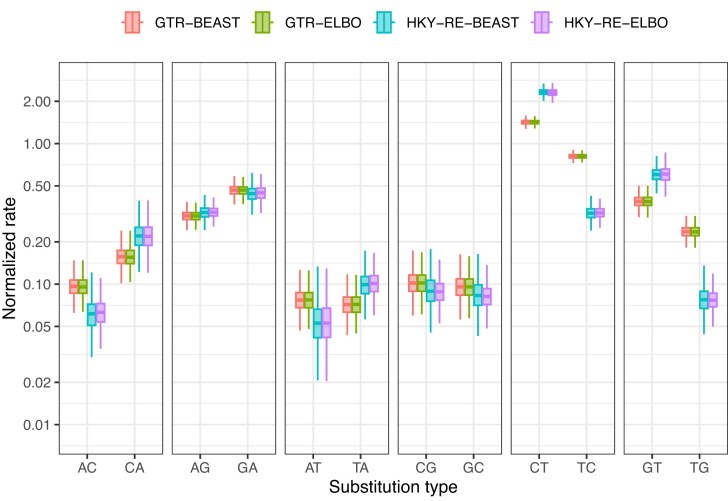
Posterior distributions of the 12 nondiagonal elements of the inferred rate matrices for the data set of Magee et al. (2024) using BEAST (MCMC) and torchtree (variational inference with ELBO). The solid line is the posterior median, and the shaded region the 50% credible interval. The whiskers extend to the posterior samples farthest from the median but within $1.5\times$ the interquartile range.

In addition to learning about how torchtree infers demographic parameters, we were motivated to learn how it infers substitution processes of a complex model. We find that the normalized substitution rates estimated using VB with torchtree closely match those obtained through BEAST (Fig. 4). Even though Magee et al. (2024) sampled the full topology space, we also find evidence for a greatly elevated rate of C$\rightarrow$T substitutions, as well as an elevated G $\rightarrow$T rate. The Bayes factor provides “very strong” (Kass and Raftery 1995) support for the nonreversibility of C$\rightarrow$T and G$\rightarrow$T rates (over the reversible model) (Table 3).

**Table 3.** Log Bayes factors favoring nonreversibility ($\operatorname{BF}_{10}$) calculated using the Savage–Dickey ratio

	Log Bayes factor	
Substitution	torchtree	BEAST
A$\leftrightarrow$C	0.9	0.6
A$\leftrightarrow$G	−0.6	−0.57
A$\leftrightarrow$T	0.52	0.4
C$\leftrightarrow$G	−0.46	−0.43
C$\leftrightarrow$T	25.9	22.08
G$\leftrightarrow$T	14.2	8.16

The ELBO- and $\operatorname{KL}(p||q)$-based approximations for the rate parameters of the substitution matrix were similar for both the GTR (Fig. S5) and HKY-RE (Figs. S7 and S8) models. However, the VB approximations using the $\operatorname{KL}(p||q)$ criterion showed slightly poorer performance when approximating some of the population size parameters (Fig. S6).

Although we expected VB to be significantly faster than MCMC, we found that this was not the case. To investigate this question, we examined how the CV evolves over time relative to the final approximation (Figs. S9 and 0S10). We found that this CV converged at similar rates for the MCMC- and VB-based analyses, although the latter is significantly more “jagged” because it represents a point estimate of the variance rather than an averaged variance. These results also suggest that the ELBO-based inference tends to converge more quickly in wall time than the $\operatorname{KL}(p||q)$-based analysis, even though the former is more computationally intensive due to gradient calculations.

## Discussion and Conclusion

The presented study introduces torchtree, a novel program for phylogenetic inference utilizing variational inference and other gradient-based algorithms. torchtree exhibits notable advantages, including enhanced speed compared with other automatic differentiation-based tools such as phylostan, and provides the capability to formulate and implement complex models and algorithms.

In our results, we highlighted that the piecewise-constant coalescent model, a nondifferentiable and discontinuous function, can lead to spurious results with ELBO-based variational inference. This is interesting because classical variational inference is based on the minimization of the $\operatorname{KL}(q||p)$ divergence, which is equivalent to maximizing the ELBO (Blei et al. 2017). Although the HMC analyses did not show similar problems for the HCV data set, there is no guarantee that analyses with other data sets will not encounter issues. We speculate that the ELBO’s mode-seeking behavior tends to get stuck in local optima introduced by discontinuities. In contrast, although discontinuities do increase the discretization error of HMC’s leapfrog integrator, these errors can be corrected through Metropolis–Hastings accept–reject steps, albeit at the cost of computational efficiency. We advocate the use of the piecewise-linear model because it has similar complexity and expressiveness to the piecewise-constant model, yet its continuous structure allows for gradient-based VB methods.

Our results showed that gradient-free variational inference with $\operatorname{KL}(p||q)$ was fast and accurate, especially for parameters of the substitution model for which gradients are notoriously expensive (Fourment et al. 2023; Magee et al. 2024). Surprisingly, our results revealed that the $\operatorname{KL}(p||q)$ objective, much like the ELBO-based estimate, accurately recovered the posterior mean but underestimated its variance. This bias is not limited to phylogenetic models; Geffner and Domke (2021) demonstrated that in high-dimensional settings, the forward KL gradient estimator tends to optimize the reverse KL. Although the estimator remains asymptotically unbiased, they suggest that the number of samples required for accurate estimation can be prohibitively large, even for simple target distributions such as a diagonal *d*-dimensional Gaussian (Geffner and Domke 2021). Increasing the number of samples would likely reduce the estimator’s bias and improve convergence, albeit at the cost of greater computation time. This underscores the need for further investigating the optimal number of samples for the SNIS estimator in phylogenetic models. To accelerate phylogenetic variational inference, a promising direction for research would be to optimize the $\operatorname{KL}(p||q)$ objective for these parameters and maximize the ELBO for the other parameters.

It is important to note that, at present, torchtree necessitates a preexisting phylogenetic tree topology as input and the topology remains fixed throughout the entire inference process. We emphasize that the fixed tree assumption fundamentally limits the accuracy of the inference, as tree inference and continuous phylogenetic model inference are intertwined.

Ongoing research is actively exploring ways to incorporate topology space exploration. This is a challenging task, as the set of phylogenetic trees is superexponential in size. MCMC avoids this problem by only exploring locally, whereas an ideal VB implementation would have a variational distribution that covers the entire space of phylogenetic trees. One approach is to restrict the support of the variational distribution using a preliminary inference step (Zhang and Matsen 2019, 2024); recent efforts to do so using supertrees (Karcher et al. 2024) and systematic search (Jennings-Shaffer et al. 2025) have not been as fruitful as hoped. Another alternative is to use a variational distribution that can potentially cover all of tree space (Koptagel et al. 2022; Mimori and Hamada 2023; Zhou et al. 2023; Sidrow et al. 2025). However, these have not yet been clearly shown to deliver reliable and efficient inference in real-world situations. Another promising effort in this vein uses a matrix representation to parameterize a large subset of tree space at a time (Bouckaert 2024).

ELBO-based inference converged more quickly to the final approximation, suggesting that incorporating gradient information in the optimization problem was beneficial, despite its computational complexity. Although variational inference is viewed as a faster alternative to MCMC, our results did not support this statement. VB appeared to approximate some parameters, such as the root height, more slowly than MCMC.

Another direction for future work is to extend beyond the mean-field variational inference used here, which is a fully factorized variational family that ignores correlation among latent variables. The full-rank approximation captures correlations between variables, but a multivariate normal distribution remains limited in its ability to represent complex interactions among latent variables. A substantial body of research model dependencies between latent variables through normalizing flows, a framework relying on a series of transformations typically learned with neural networks (Rezende and Mohamed 2015). Although Zhang (2020) reported some promising results, Ki and Terhorst (2022) suggested that normalizing flows did not yield improvements in the approximation. This discrepancy may stem from the substantial differences in the phylogenetic models explored by these researchers. Our preliminary analyses with normalizing flow have also shown no improvement in the final approximation (data not shown). A deeper investigation into normalizing flows is warranted, as well as exploration of other methods that build structured variational families (Yin and Zhou 2018; Ambrogioni et al. 2021).

Overall, we believe that torchtree is a valuable step toward more flexible modeling in phylogenetics, enabling rapid model development without the need to derive gradients analytically and allowing the use of gradient-based algorithms such as HMC, VB, and Laplace approximation. This is made possible by leveraging a framework that provides automatic differentiation (in this case, PyTorch), enabling the rapid development of new models and algorithms. Indeed, as many cutting-edge model architectures are already implemented in PyTorch, they are now immediately available for phylogenetic inference.

## Supplementary Material

syaf047_Supplemental_File
