# Supplementary material for: torchtree: Flexible Phylogenetic Model Development and Inference Using PyTorch
Source: Syst Biol. 2025 Jul 4;75(1):39–51. doi: 10.1093/sysbio/syaf047 (PMC12805669; doi:10.1093/sysbio/syaf047)
Supplement: syaf047_Supplemental_File [file syaf047_supplemental_file.pdf]

Supplementary Material

1 Methods

1.1 Mass-Covering vs. Mode-Seeking: Forward vs. Reverse KL

In this section we illustrate using a simple example the difference between reverse and forward Kullback-Liebler (KL) by optimizing the ELBO and the self-normalizing importance sampling estimator L_{KL}, respectively.

We define the target distribution as a mixture of two normal distributions:

$$p(x) = \sum_{k=1}^{K} \pi_k \mathcal{N}(x; \mu_k, \sigma_k^2)$$

where $K = 2$, $\mu_1 = 0$, $\mu_2 = 10$, $\sigma_1^2 = \sigma_2^2 = 1$ and $\pi_1 = \pi_2 = 0.5$.

The variational distribution is a univariate normal distribution, which of course is ill-suited for a bimodal distribution, but this simple example illustrates the difference between mode-seeking and mass-covering of the two different KL divergence The gradient of the ELBO is calculated with 1 sample while the gradient L_{KL} is calculate with different number of samples (1, 10, 100, 1000).

The mode-seeking behaviour of $\text{KL}(q||p)$ is evident in Supplementary Figure 1, where the variational distribution collapses onto a single mode. In contrast, the mass-covering tendency of $\text{KL}(p||q)$ results in a variational distribution that spreads across most of the target distribution, capturing both modes.

1.2 Continuity and differentiability of piecewise-linear coalescent model

A piecewise-linear function consists of multiple linear segments joined at specific points, known as "breakpoints." Although each segment is linear, the overall function is continuous if it does not have any abrupt jumps at the breakpoints. In a continuous function, the limit from the left (as you approach a point from the left side) and the limit from the right (as you approach a point from the right side) must be equal to the value of the function at that point.

For $M \geq 3$ segments, two adjacent segments that do not include the last segment are defined as

$$\widehat{N}(t) = \begin{cases} \theta_i + (\theta_{i+1} - \theta_i)\frac{t - x_i}{x_{i+1} - x_i} & \text{if } x_i \leq t \leq x_{i+1} \\ \theta_{i+1} + (\theta_{i+2} - \theta_{i+1})\frac{t - x_{i+1}}{x_{i+2} - x_{i+1}} & \text{if } x_{i+1} \leq t \leq x_{i+2} \end{cases}.$$

These segments are continuous since $\lim_{t \to x_{i+1}^-} \widehat{N}(t) = \lim_{t \to x_{i+1}^+} \widehat{N}(t) = \widehat{N}(x_{i+1}) = \theta_{i+1}$.

For the last two segments we have

$$\widehat{N}(t) = \begin{cases} \theta_M + (\theta_M - \theta_{M-1})\frac{t - x_{M-1}}{x_M - x_{M-1}} & \text{if } x_{M-1} \leq t \leq x_M \\ \theta_M & t \geq \text{if } x_M \end{cases}.$$

The last two segments are also continuous since $\lim_{t \to x_M^-} \widehat{N}(t) = \lim_{t \to x_M^+} \widehat{N}(t) = \widehat{N}(x_M) = \theta_M$.

The piecewise-linear model is continuous since $\lim_{t \to x_{i+1}^-} \widehat{N}(t) = \lim_{t \to x_{i+1}^+} \widehat{N}(t) = \widehat{N}(x_{i+1})$ at every boundary.

A function is differentiable at a point if it has a well-defined, finite derivative at that point. For a piecewise-linear function, differentiability requires that the function be both continuous and have matching derivatives from the left and right at each breakpoint.

$$\widehat{N}'(t) = \begin{cases} \frac{\theta_{i+1} - \theta_i}{x_{i+1} - x_i} & \text{if } x_i \leq t \leq x_{i+1} \\ \frac{\theta_{i+2} - \theta_{i+1}}{x_{i+2} - x_{i+1}} & \text{if } x_{i+1} \leq t \leq x_{i+2} \end{cases}.$$

The function is not differentiable since $\lim_{t \to x_{i+1}^-} \widehat{N}'(t) \neq \lim_{t \to x_{i+1}^+} \widehat{N}'(t)$.

1.3 Validation of piecewise-linear coalescent model

We examine the ability of the piecewise-linear coalescent model to recover effective population size dynamics in a simulation study. First, we simulate a phylogeny assuming a constant population size $N_e(t) = 1.0$. Next, we simulate a phylogeny assuming a linear function with $N_e(0) = 0.1$ and the slope is defined such that $N_e(t_0) = 1.0$ where t_0 is the age of the root. Finally, we simulate a phylogeny simulated with a bottleneck during the population's evolutionary history. We define the piecewise function as

$$N_e(t) = \begin{cases} 1 & t \leq 4 \\ 0.1 & 4 < t < 6 \\ 1 & t \geq 6. \end{cases}$$

Every simulated phylogeny contains 500 serially sampled taxa. We either used a piecewise-constant (aka skygrid) [Gill et al., 2013] or piecewise-linear (skyglide) population size coalescent prior with a cutoff equal to the root height and 100 time segments. As in the HCV analysis, we place a Gaussian Markov random field (GMRF) prior on the vector of log effective population sizes and a gamma prior with rate and scale equal to 0.005 on the precision parameter.

The simulation and inference code can be found online `https://github.com/4ment/torchtree-notebooks/blob/main/notebooks/skyglide_validation.ipynb`.

1.4 Bayes factor calculation for meanfield variational inference

Following the notation of Magee et al. [2024], the Bayes factor in favor of $\Delta_{ij} = 0$ (Model 0, against Model 1 where Δ_{ij} is a free parameter) is the ratio of the posterior density to the prior density at $\Delta_{ij} = 0$,

$$\mathrm{BF}_{01} = \frac{p(\Delta_{ij} = 0|y)}{p(\Delta_{ij} = 0)} \approx \frac{q^*(\Delta_{ij} = 0)}{p(\Delta_{ij} = 0)},$$

where q^* is a normal distribution with mean $m_{ji} - m_{ij}$ and variance $\sigma^2_{ij} + \sigma^2_{ji}$. m_{ij} and σ_{ij} are the mean and standard deviation of the normal variational approximation of ϵ_{ij}. The Bayes factor favoring nonreversibility ($\Delta_{ij} \neq 0$) is $\mathrm{BF}_{10} = 1/\,\mathrm{BF}_{01}$. [Magee et al., 2024] provide a closed-form expression for the probability density of $\Delta_{ij} = 0$ evaluated at 0

$$p(\Delta_{ij} = 0) = \frac{\alpha\Gamma(\delta + 1/\alpha)}{2^{1+1/\alpha}\Gamma(1/\alpha)\Gamma(\delta)\beta^{1/\alpha}}.$$

2 Results

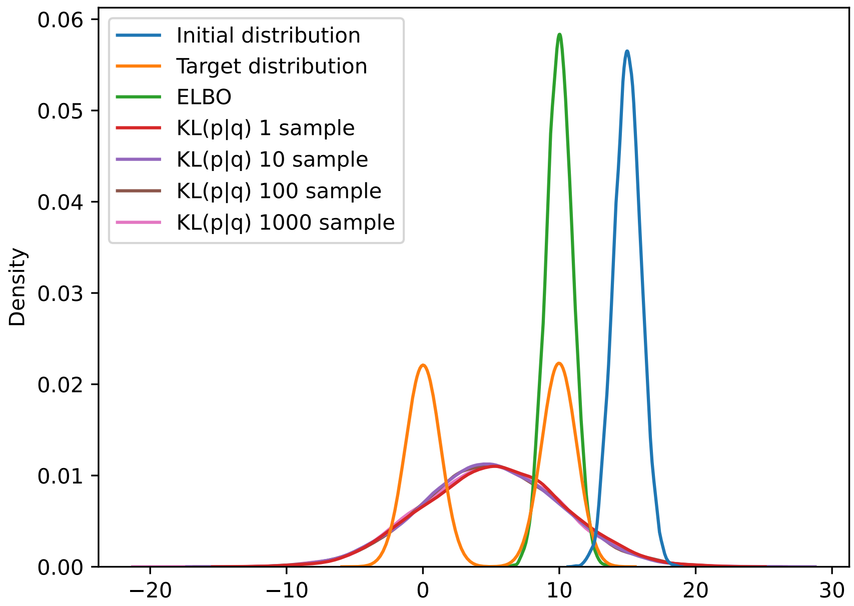

Figure 1: Results of optimizing the KL($q||p$) (ELBO) vs. KL($p||q$) for a normal variational approximation on a bimodal target. In every analysis, the variational distribution is initialized with $\mu = 15$ and $\sigma = 2$ (density labelled *Initial distribution*).

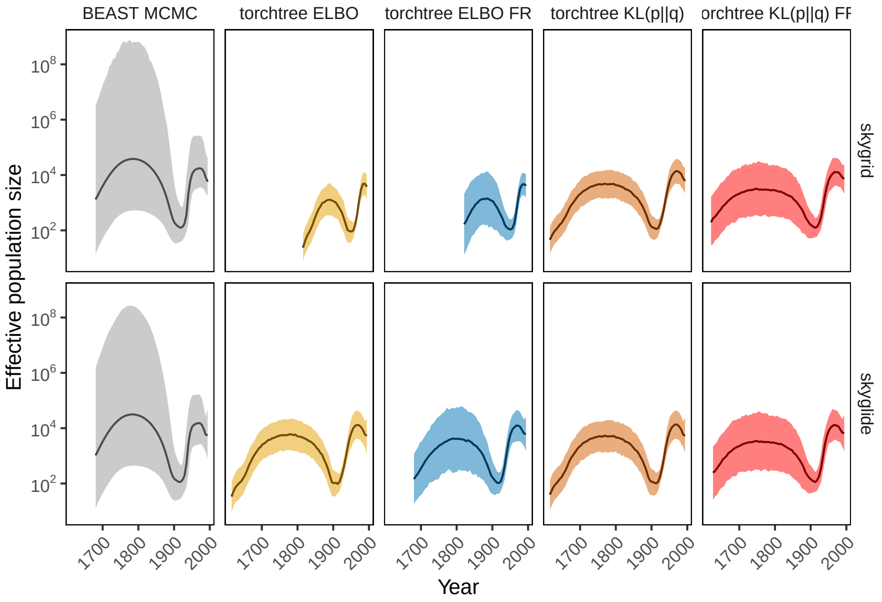

Figure 2: Posterior approximation of skyglide (piecewise-linear) and skygrid (piecewise-constant) population size distributions using `torchtree` and BEAST on the HCV dataset. `torchtree` approximates the distributions using either mean-field or full-rank (label ending with FR) variational inference (ELBO and KL($p||q$)).

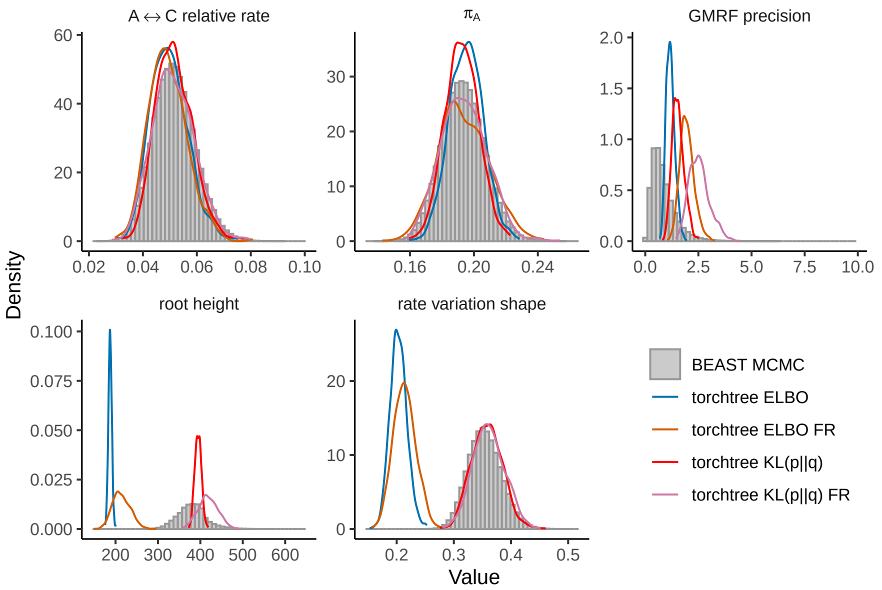

Figure 3: Posterior approximation of phylogenetic model parameters using `torchtree` and BEAST on the HCV dataset with the skygrid (piecewise-constant) model. `torchtree` approximates the distributions using either mean-field or full-rank (label ending with FR) variational inference (ELBO and KL($p||q$)). BEAST uses MCMC. The plot displays density distributions for several parameters: the substitution rate bias between nucleotide A and C (A $\leftrightarrow$ C), the frequency of nucleotide A (π_{A}), the GMRF precision parameter, the age of the root node (root height) and the shape parameter of the discrete gamma site model. The gradient-based ELBO inference clearly struggles in this case of a discontinuous model.

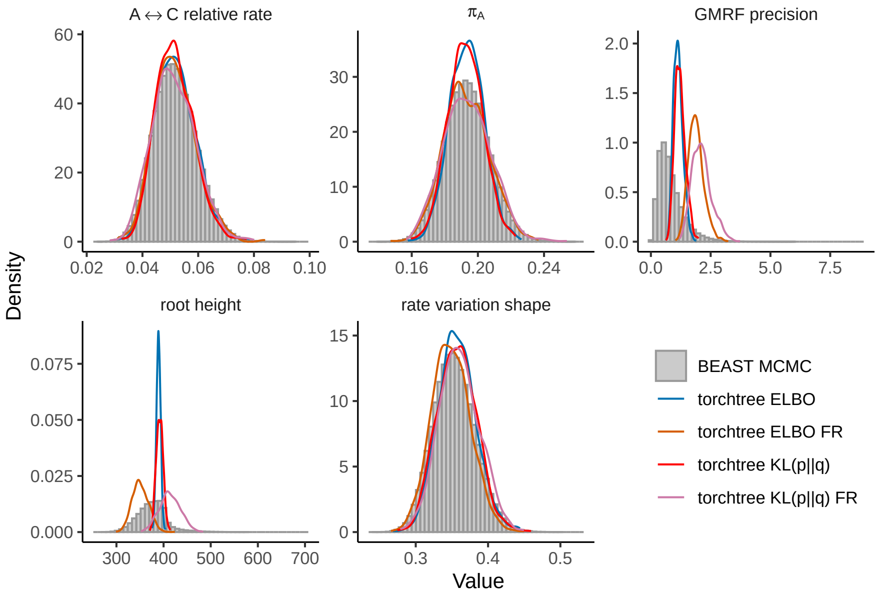

Figure 4: Posterior approximation of phylogenetic model parameters using `torchtree` and BEAST on the HCV dataset with the skyglide (piecewise-linear) model. `torchtree` approximates the distributions using either mean-field or full-rank (label ending with FR) variational inference (ELBO and KL($p||q$)). BEAST uses MCMC. The plot displays density distributions for several parameters: the substitution rate bias between nucleotide A and C (A $\leftrightarrow$ C), the frequency of nucleotide A (π_A), the GMRF precision parameter, the age of the root node (root height) and the shape parameter of the discrete gamma site model.

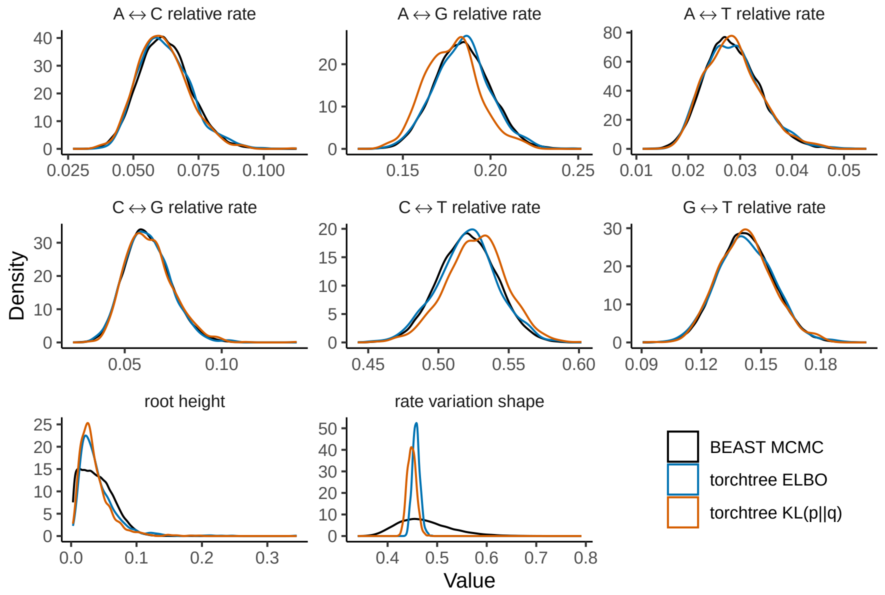

Figure 5: Posterior approximation of phylogenetic model parameters using `torchtree` and BEAST on the SC2 dataset with the GTR substitution model. `torchtree` approximates the distributions using variational inference with either the ELBO or KL($p||q$) objective functions, whereas BEAST employs MCMC. The plot displays density distributions for several parameters: the substitution rate bias parameters ($\mathrm{A} \leftrightarrow \mathrm{C}, \mathrm{A} \leftrightarrow \mathrm{G}, \ldots, \mathrm{G} \leftrightarrow \mathrm{T}$), the age of the root node (root height) and the shape parameter of the discrete gamma site model.

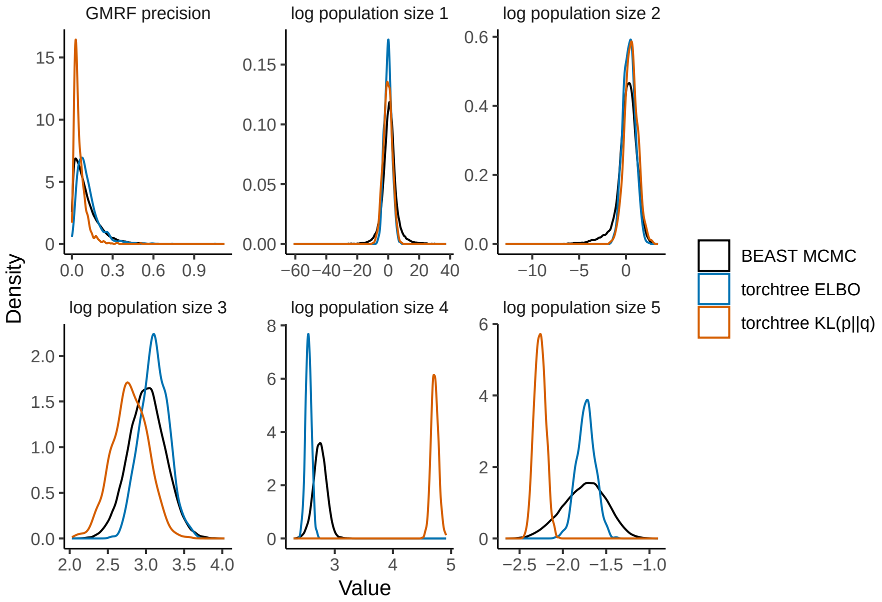

Figure 6: Posterior approximation of the parameters of the coalescent and Gaussian Markov random field (GMRF) models using `torchtree` and BEAST on the SC2 dataset with the GTR substitution model. `torchtree` approximates the distributions using variational inference with either the ELBO or KL$(p||q)$ objective functions, whereas BEAST employs MCMC. The plot displays density distributions for the five population size parameters and the precision parameter of the GMRF.

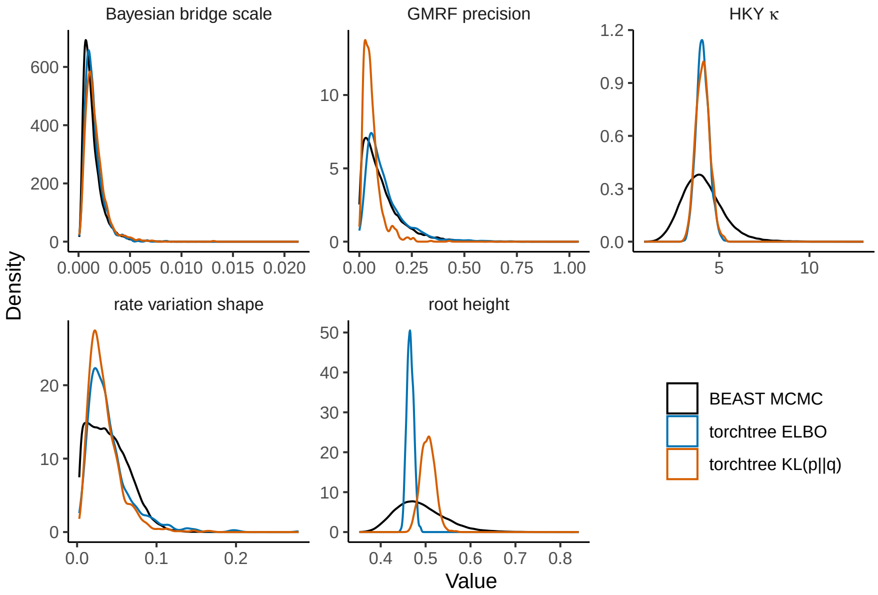

Figure 7: Posterior approximation of phylogenetic model parameters using `torchtree` and BEAST on the SC2 dataset with the HKY-RE substitution model. `torchtree` approximates the distributions using variational inference with either the ELBO or KL($p||q$) objective functions, whereas BEAST employs MCMC. The plot displays density distributions for several parameters: the scale parameter of the Bayesian bridge prior, the precision parameter of the GMRF, the ratio of transition and transversion rate parameter (HKYκ) of the HKY model, the shape parameter of the discrete gamma site model, and the age of the root node.

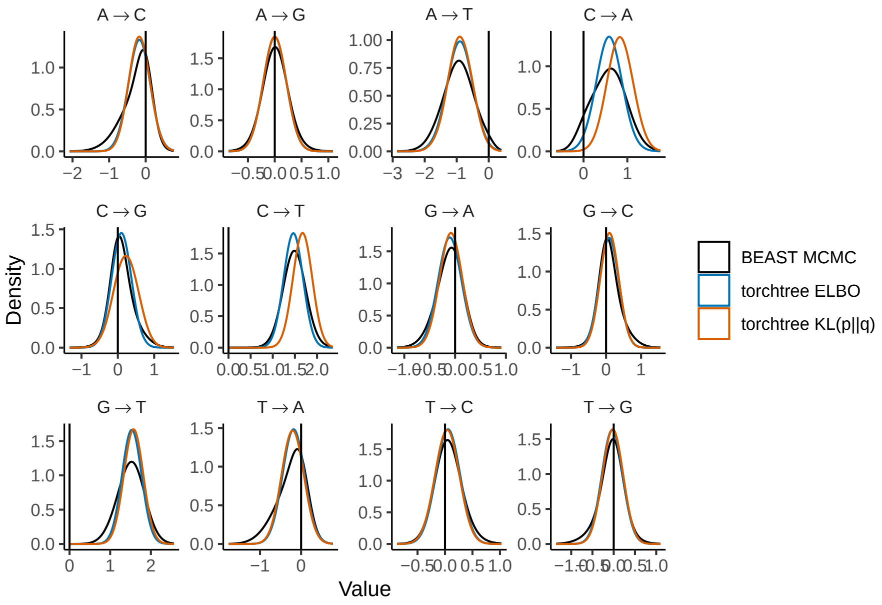

Figure 8: Posterior approximation of the random effect parameters using `torchtree` and BEAST on the SC2 dataset with the HKY-RE substitution model. `torchtree` approximates the distributions using mean-field variational inference (ELBO and KL($p||q$)) and BEAST uses MCMC.

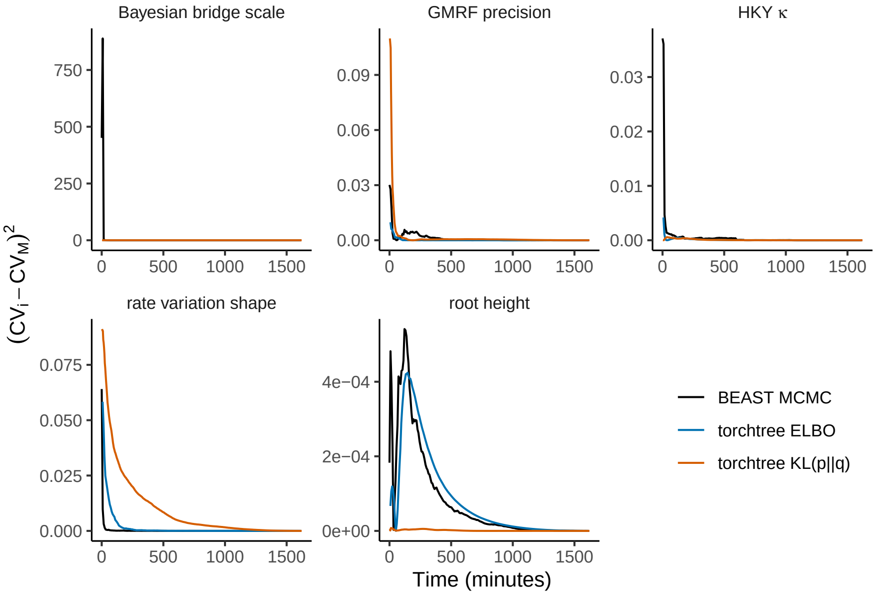

Figure 9: Convergence between intermediate estimates and the final approximation of phylogenetic model parameters using `torchtree` and BEAST on the SC2 dataset with the HKY-RE substitution model. `torchtree` approximates the distributions using variational inference with either the ELBO or KL$(p||q)$ objective functions, whereas BEAST employs MCMC. The y-axis represents the squared difference between the coefficient variation (CV) at time i and the CV of the final approximation at time M while the x-axis represent time.

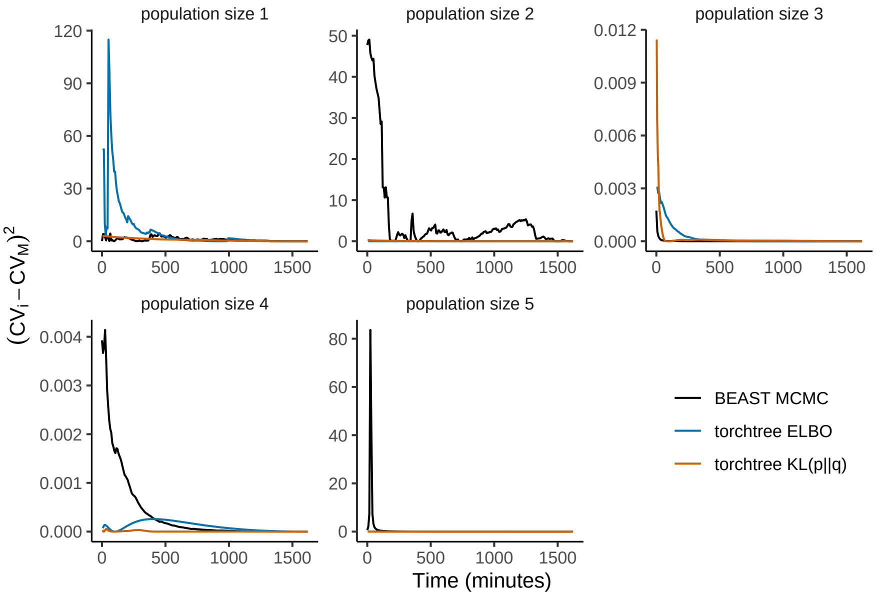

Figure 10: Convergence between intermediate estimates and the final approximation of the five population size parameters using `torchtree` and BEAST on the SC2 dataset with the HKY-RE substitution model. `torchtree` approximates the distributions using variational inference with either the ELBO or KL($p||q$) objective functions, whereas BEAST employs MCMC. The y-axis represents the squared difference between the coefficient variation (CV) at time i and the CV of the final approximation at time M while the x-axis represent time.

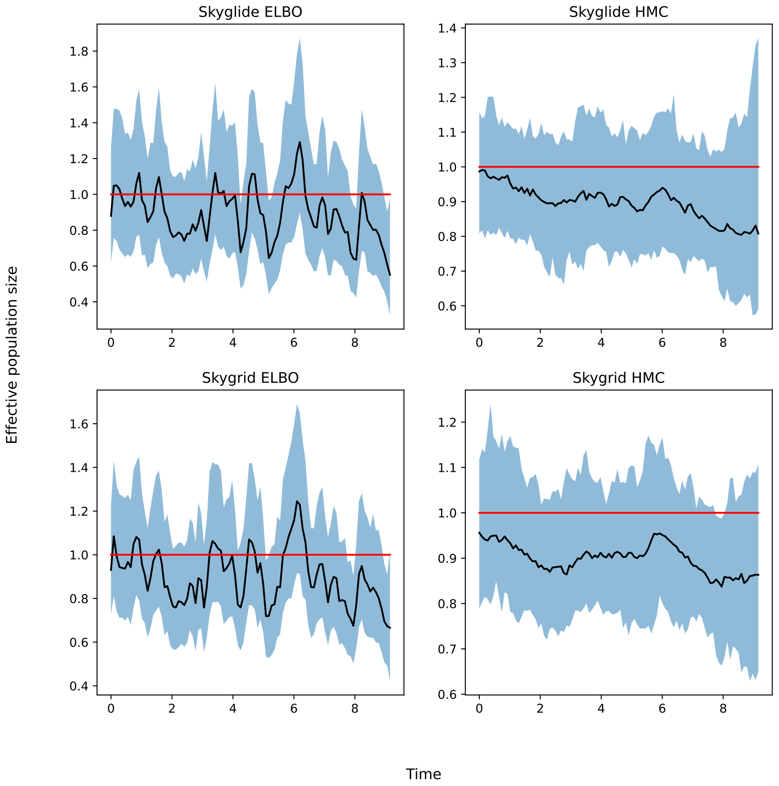

Figure 11: Posterior approximation of skyglide (piecewise-linear) and skygrid (piecewise-constant) distributions using `torchtree` on a phylogeny simulated assuming a constant population size. The plots show posterior median (solid black line) and 95% Bayesian credible intervals (blue shading) of the effective population size. The red lines represent the true population size trajectory that was used for simulations.

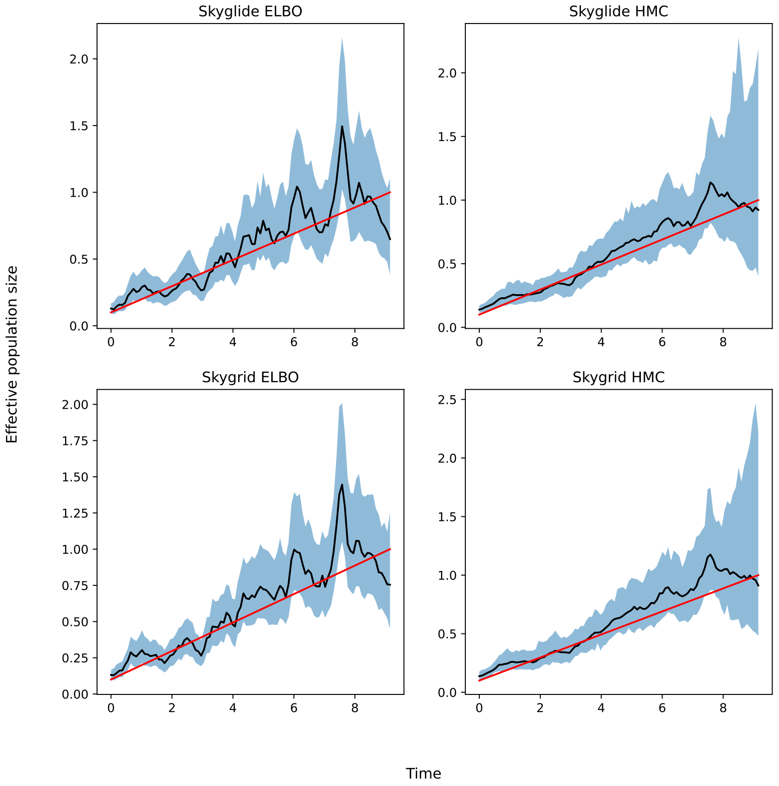

Figure 12: Posterior approximation of skyglide (piecewise-linear) and skygrid (piecewise-constant) distributions using `torchtree` on a phylogeny simulated assuming a linearly increasing population size. The plots show posterior median (solid black line) and 95% Bayesian credible intervals (blue shading) of the effective population size. The red lines represent the true population size trajectory that was used for simulations.

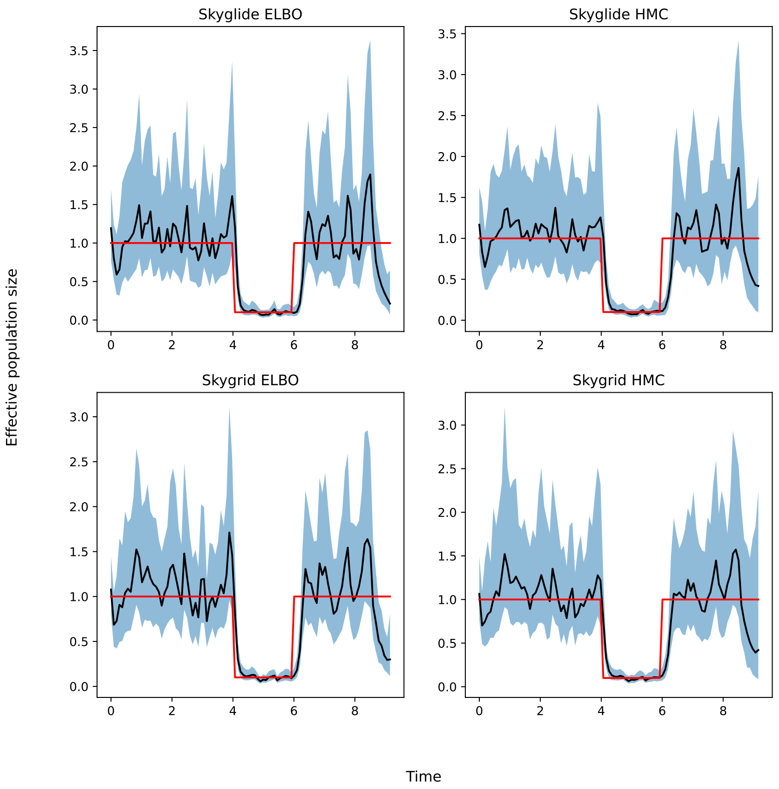

Figure 13: Posterior approximation of skyglide (piecewise-linear) and skygrid (piecewise-constant) distributions using `torchtree` on a phylogeny simulated with a bottleneck during the population's evolutionary history. The plots show posterior median (solid black line) and 95% Bayesian credible intervals (blue shading) of the effective population size. The red lines represent the true population size trajectory that was used for simulations.

References

Mandev S Gill, Philippe Lemey, Nuno R Faria, Andrew Rambaut, Beth Shapiro, and Marc A Suchard. Improving Bayesian population dynamics inference: a coalescent-based model for multiple loci. *Molecular biology and evolution*, 30 (3):713–724, 2013.

Andrew F Magee, Andrew J Holbrook, Jonathan E Pekar, Itzue W Caviedes-Solis, Fredrick A Matsen IV, Guy Baele, Joel O Wertheim, Xiang Ji, Philippe Lemey, and Marc A Suchard. Random-effects substitution models for phylogenetics via scalable gradient approximations. *Systematic Biology*, 73(3): 562–578, 05 2024. ISSN 1063-5157. doi: 10.1093/sysbio/syae019. URL `https://doi.org/10.1093/sysbio/syae019`.
